# Congestion assessment using a self-supervised contrastive learning-derived risk index in patients with congestive heart failure (CONAN): protocol and design of a prospective cohort study

**DOI:** 10.1093/ehjdh/ztaf004

**Published:** 2025-08-04

**Authors:** Bojan Hartmann, Niels-Ulrik Hartmann, Julia Brandts, Marlo Verket, Nikolaus Marx, Niveditha Dinesh, Lisa Schuetze, Anna Emilia Pape, Dirk Müller-Wieland, Markus Kollmann, Katharina Marx-Schütt, Martin Berger, Andreas Puetz, Felix Michels, Luca Leon Happel, Lars Müller, Guido Kobbe, Malte Jacobsen

**Affiliations:** Department of Cardiology, Angiology and Intensive Care Medicine, Medical Faculty, RWTH Aachen University, Pauwelsstraße 30, Aachen 52074, Germany; Department of Cardiology, Angiology and Intensive Care Medicine, Medical Faculty, RWTH Aachen University, Pauwelsstraße 30, Aachen 52074, Germany; Department of Cardiology, Angiology and Intensive Care Medicine, Medical Faculty, RWTH Aachen University, Pauwelsstraße 30, Aachen 52074, Germany; Department of Cardiology, Angiology and Intensive Care Medicine, Medical Faculty, RWTH Aachen University, Pauwelsstraße 30, Aachen 52074, Germany; Department of Cardiology, Angiology and Intensive Care Medicine, Medical Faculty, RWTH Aachen University, Pauwelsstraße 30, Aachen 52074, Germany; Department of Cardiology, Angiology and Intensive Care Medicine, Medical Faculty, RWTH Aachen University, Pauwelsstraße 30, Aachen 52074, Germany; Department of Cardiology, Angiology and Intensive Care Medicine, Medical Faculty, RWTH Aachen University, Pauwelsstraße 30, Aachen 52074, Germany; Department of Cardiology, Angiology and Intensive Care Medicine, Medical Faculty, RWTH Aachen University, Pauwelsstraße 30, Aachen 52074, Germany; Department of Cardiology, Angiology and Intensive Care Medicine, Medical Faculty, RWTH Aachen University, Pauwelsstraße 30, Aachen 52074, Germany; Department of Biology, Heinrich Heine University Düsseldorf, Düsseldorf 40225, Germany; Department of Cardiology, Angiology and Intensive Care Medicine, Medical Faculty, RWTH Aachen University, Pauwelsstraße 30, Aachen 52074, Germany; Department of Cardiology, Angiology and Intensive Care Medicine, Medical Faculty, RWTH Aachen University, Pauwelsstraße 30, Aachen 52074, Germany; Department of Cardiology, Angiology and Intensive Care Medicine, Medical Faculty, RWTH Aachen University, Pauwelsstraße 30, Aachen 52074, Germany; Department of Biology, Heinrich Heine University Düsseldorf, Düsseldorf 40225, Germany; Department of Haematology, Oncology, and Clinical Immunology, Medical Faculty, University Hospital Düsseldorf, Heinrich Heine University Düsseldorf, Düsseldorf 40225, Germany; Department of Haematology, Oncology, and Clinical Immunology, Medical Faculty, University Hospital Düsseldorf, Heinrich Heine University Düsseldorf, Düsseldorf 40225, Germany; Department of Haematology, Oncology, and Clinical Immunology, Medical Faculty, University Hospital Düsseldorf, Heinrich Heine University Düsseldorf, Düsseldorf 40225, Germany; Department of Cardiology, Angiology and Intensive Care Medicine, Medical Faculty, RWTH Aachen University, Pauwelsstraße 30, Aachen 52074, Germany

**Keywords:** Congestive heart failure, Acute decompensated heart failure, Wearable, Remote patient monitoring, Self-supervised contrastive learning

## Abstract

**Aims:**

Recurrent congestive episodes are a primary cause of hospitalizations in patients with heart failure. Hitherto, outpatient management adopts a reactive approach, assessing patients clinically through frequent follow-up visits to detect congestion early. This study aims to assess the capabilities of a self-supervised contrastive learning-derived risk index to detect episodes of acute decompensated heart failure (ADHF) in patients using continuously recorded wearable time-series data.

**Methods and results:**

This is the protocol for a single-arm, prospective cohort pilot study that will include 290 patients with ADHF. Acute decompensated heart failure is diagnosed by clinical signs and symptoms, as well as additional diagnostics (e.g. NT-proBNP). Patients will receive standard-of-care treatment, supplemented by continuous wearable-based monitoring of vital signs and physical activity, and are followed for 90 days. During follow-up, study visits will be conducted and presentations without clinical ADHF will be referred to as ‘regular’ and data from these episodes will be presented to a deep neural network that is trained by a self-supervised contrastive learning objective to extract features from the time-series that are typical in regular periods. The model is used to calculate a risk index measuring the dissimilarity of observed features from those of regular periods. The primary outcome of this study will be the risk index’s accuracy in detecting episodes with ADHF. As secondary outcome data integrity and the score in the validated questionnaire System Usability Scale will be evaluated.

**Conclusion:**

Demonstrating reliable congestion detection through continuous monitoring with a wearable and self-supervised contrastive learning could assist in pre-emptive heart failure management in clinical care.

**Clinical trial registration:**

The study was registered in the German clinical trials register (DRKS00034502).

## Graphical Abstract

**Graphical Abstract:**
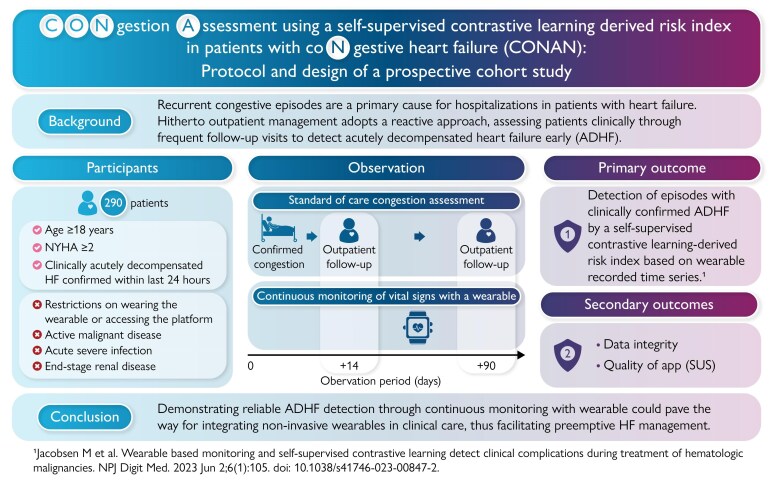

## Introduction

Heart failure (HF) is the most common cause of hospitalization and in-hospital death in Germany.^1^ After discharge, 17% of patients with HF are rehospitalized for HF within 90 days.^2^ Recurrent hospitalizations of respective patients due to HF increase the risk of adverse events, and these are associated with high mortality rates of almost 12%.^3,4^ Up to 70% of HF-related hospitalizations are driven by acute decompensated HF (ADHF).^5^ Acute decompensated HF can be triggered by various mechanisms such as hypertensive episodes or arrhythmias.^6,7^ Hitherto, outpatient management of HF patients relies on self-assessment of symptoms and tracking of single parameters like body weight. This approach places a significant burden on patients, requiring them to handle standardized parameters and possess a certain level of health literacy.^8^ To reduce the risk of hospitalizations, patients with HF are frequently re-examined and clinically assessed according to fixed schedules to detect decompensation early.^9^ These visits involve clinical assessment with specialized personnel, laboratory measurements, and adjunctive diagnostic modalities, which are resource-intensive and pose a significant burden to specialized outpatient clinics.^10^ Hence, there is a need for innovative and individualized concepts for pre-emptive detection of congestion in patients with HF that reduce the burden on the healthcare system.

Implementation of digital tools for assessing early clinical signs of congestion represents an option for the management of patients with HF and potentially facilitates early treatment interventions.^11^ Cardiac implantable electronic devices and implantable pulmonary artery pressure sensors are promising methods for detecting congestion early; however, they require an invasive implantation procedure.^11,12^ Devices measuring bioelectrical impedance show comparable results in detecting congestion; however, they require active patient engagement.^13^

Small, portable devices known as ‘wearables’ offer a novel option for non-invasive monitoring of vital parameters and physical activity.^14^ These devices utilize established sensor technologies such as photoplethysmography, accelerometry, and temperature probes to continuously record data about relevant parameters for medical monitoring. However, despite their potential, wearables are predominantly used for sports and lifestyle monitoring, with medical applications remaining relatively scarce. This scarcity is primarily due to the challenge of extracting clinically actionable information, or digital biomarkers, from the extensive data generated by wearables. A study with multi-parameter adhesive chest wearables and advanced analytics showed good accuracy in detecting impending hospitalizations but revealed adherence issues that may hinder the widespread clinical application of wearables.^15^ There is a need for unobtrusive systems that enable longitudinal and passive monitoring of patients with HF.

In a previous study, we demonstrated the feasibility of detecting clinical complications such as infections and arrhythmias leveraging wearable data obtained in oncology patients undergoing chemotherapy by computing a risk index using deep learning analysis (‘self-supervised contrastive learning’).^16^

In this study, the aim is to evaluate the accuracy of a similar risk index in detecting ADHF in patients with HF, assess the data integrity, and explore the patients’ perspective.

## Methods

### Study design and setting

This is an investigator-initiated, single-centre, single-arm, prospective cohort pilot study including patients with ADHF. The study will be conducted at the Department of Cardiology, Angiology, and Intensive Care Medicine of the University Hospital RWTH Aachen. The study was approved by the ethics committee of the Medical Faculty of University Hospital RWTH Aachen and was registered in the German clinical trials register (DRKS00034502). This study will be conducted according to the Helsinki Declaration.

Patients will be informed that they will not derive immediate individual benefits from study participation. All patients will sign a written informed consent before study inclusion. Recruitment will start in December 2024, and study duration is planned for 24 months. There will be no study-related follow-up of the patients after completion of the study.

### Participants

Inclusion criteria will be patients’ age ≥ 18 years with ADHF confirmed within the last 24 h. Acute decompensated HF is defined as worsening in dyspnoea at rest or with minimal activity, one or more accompanying signs (jugular venous pulsation ≥ 11 cm, hepatomegaly, peripheral oedema), and one or more objective measures of heart failure (six minute walking test < 200 m, N-terminal proBNP level ≥ 1000 pg/mL or a two-fold increase to baseline).^17^ Additionally, one of the following findings has to apply: chest X-ray (presence of pulmonary venous congestion or small pleural effusion) or vena cava imaging (one of two max. diameter > 2.2 cm and/or collapsibility < 50%) or lung ultrasound (more than three B-lines in more than two intercostal spaces bilaterally are considered diagnostic for the detection of interstitial and alveolar oedema). Exclusion criteria are defined as active malignant disease, pregnancy, active severe systemic infection (requiring intravenous antibiotic treatment), end-stage renal disease with dialysis, medical or mental conditions impairing the ability to continuously wear the wearable (e.g. dementia, skin abnormalities), and active implants, which might impair recordings (detailed inclusion and exclusion criteria are listed in *Table 1*).

**Table 1:** Inclusion and exclusion criteria for the CONAN study

Inclusion criteria	Exclusion criteria
Clinically confirmed congestion within the last 24 h NYHA ≥ II Age ≥ 18 years and able to understand the design and objectives of the study Signed written informed consent and data safety agreement before any study-related activities Willingness to wear the wearable during the treatment period and return it on a follow-up visit	Active malignant disease Pregnancy Active severe systemic infection, defined as intravenous administration of antibiotics End-stage renal disease with dialysis Medical or mental conditions, such as dementia, that hinder the continuous use of the monitoring platform and associated equipment during the monitoring period Any condition that makes wearing the wearable device impossible (e.g. presence of a shunt, active bleeding, or absence of limbs) Mental incapacity or language barriers that preclude adequate understanding or cooperation, known or suspected not to comply with study directives or not to be reliable or trustworthy, or a subject who in the opinion of the investigator should not participate in the study

### Study procedures

**Figure 1:**
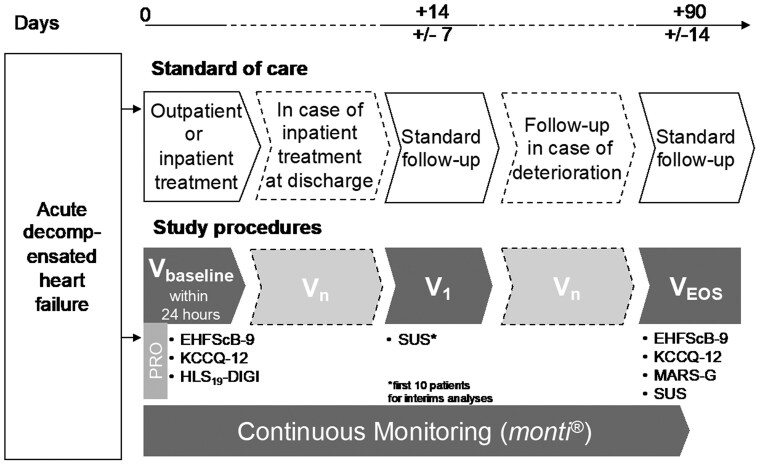
During the CONAN study, clinical congestion assessment will be conducted at study visits. These procedures will be consistently repeated at each visit. Subsequently, clinical endpoints will be evaluated.

In- and outpatients with ADHF will consecutively be screened for in- and exclusion criteria at the cardiology department at University Hospital RWTH Aachen (*Figure 1*). All patients will be treated according to the standard of care, and the study visits will be scheduled accordingly. The monitoring period starts from the inclusion of the patients into the study followed by continuous monitoring of vital signs and physical activity by the wearable for 90 days. Based on the decision by the treating physician eligible patients will be admitted for treatment to the study site or will be treated as outpatients. Follow-up visits are conducted on the day of discharge and in accordance with the guideline-based follow-up recommendations for patients experiencing ADHF, routine outpatient visits at the HF clinic will be scheduled ∼14 and 90 days post-discharge. If the patient initially receives inpatient treatment, the day of discharge and any unplanned visit to the outpatient clinic of the study site will be documented as study visits.

The baseline visit of a given patient will be conducted within the initial 24 h after diagnosis of ADHF. During the baseline visit, the following data will be obtained: medical history including HF entity, comorbidities, symptoms, signs, vital signs, laboratory values, and bioelectrical impedance analysis (*Table 2*). Bioelectrical impedance analysis will be performed by clinical staff to assess the patient’s total body water composition. Additionally, the EVEREST Score will be computed that serves as an established clinical tool for assessing congestion in patients with HF. The score scales rate congestion severity, utilizing clinical parameters such as dyspnoea, orthopnoea, oedema, and jugular venous pressure.^18^ Three paper-based validated questionnaires will be used to assess digital health literacy (HLS_19_-DIGI) overall, self-care behaviour in HF using the nine-item European Heart Failure Self-care Behaviour Scale (EHFScB-9), and the Kansas City Cardiomyopathy Questionnaire (KCCQ-12), which assesses the quality of life in patients with HF.

**Table 2:** Study procedures during the CONAN study

Procedures	Baseline (V_Baseline_) Confirmed ADHF Day 0/+24 h	Visit a (V_a_) In the case of inpatient treatment, the day of discharge	Visit 1 (V_1_) Regular outpatient visit Day +14 days (±7 days)	Visit *n* (V*_n_*) Any unplanned clinic visit (i.e. ER visit) Day +*n*	Visit end-of-study (V_EOS_) Regular outpatient visit Day +90 days ± 14 days
Inclusion/exclusion criteria	X				
Demographics	X				
Past medical history incl. HF entity	X				
Concomitant medication	X	X	X	X	
Vital signs and body weight	X	X	X	X	X
HLS_19_-DIGI	X				
Kansas City Cardiomyopathy Questionnaire (KCCQ-12)	X				X
European Heart Failure Self-care Behaviour Scale (EHFScB-9)	X				X
Safety/Deficiencies Assessment		X	X	X	
EVEREST Score^18^	X	X	X	X	X
Mobile Application Rating Scale (MARS-G)^19^					X
System Usability Scale (SUS)					X
Bioelectrical impedance	X	X	X	X	X
Parameters using the *monti* platform					
Connection attempts to the server		X	X	X	X
Number of daily ECG recordings		X		X	X
Completed uploads including timestamp and file size		X	X	X	X
Failed uploads		X	X		X
Completeness of vital signs and physical activity time-series data:		X	X		X
Number of notifications and type (e.g. reactivate/charging)		X	X		X
Number and type of contacts (e.g. phone call for technical support)					X

During follow-up visits, symptoms, signs, and vital signs will be assessed. As part of the safety assessments, local skin reactions or discomfort are monitored, and the technical equipment is inspected for any obvious damage. Moreover, details regarding HF hospitalizations and all unplanned, non-trauma-related hospitalizations and emergency department visits will be recorded. During the visit at 14 days post-discharge, the first 10 patients will complete the System Usability Scale (SUS) to identify any potential deficits in the monitoring platform that can be iteratively optimized. The SUS measures the overall usability of a system by capturing user perceptions of its ease of use and learnability. As there are multiple translations to German, the version of Rummel *et al*. was chosen.^20^

The end-of-study visit will be scheduled on Day 90 post-initial discharge, with a window of plus or minus 14 days. EHFScB-9 and KCCQ-12 questionnaires are completed by the patients again. Additionally, the SUS and Mobile App Rating Scale (MARS-G) questionnaires will be handed out to the patients. These are validated tools used to assess the quality and usability of digital health applications.^19^ MARS-G evaluates various aspects of mobile app performance, including engagement, functionality, aesthetics, and information quality. In the event of withdrawal of consent, patients will be asked to complete an end-of-study visit at the earliest possible date.

### Wearable-based monitoring platform

We developed ‘monti’ a platform that continuously records vital and activity parameters in near real-time using a non-invasive wearable, securely transmits the data, and analyses it with self-learning artificial intelligence. Enrolled patients will receive a registered smartphone and the CardioWatch 287-2 wrist-wearable (Corsano Health B.B., Isaac da Costalaan 20, 1401 BH Bussum, The Netherlands). The smartphone will function both as a wearable data proxy and a user interface for the assessment of electronic patient-reported outcomes (ePRO) via a smartphone application. The commercially available wearable employed is a CE-marked medium-risk device (class IIa) according to Directive 93/42/EEC.^21^ Various sensors integrated into this wearable device are utilized for monitoring vital signs and physical activity, including photoplethysmography, temperature probe, and accelerometer (*Table 3*). The electrocardiogram (ECG) sensor necessitates active patient engagement, with patients requested to record a 30 s ECG once daily. Patients will be instructed to charge the wearable every other day for ∼90 min. Additionally, a brief patient manual with key information and contact will be provided. With the app, patients can record ePRO regarding their subjective symptoms in a concise symptom inventory. The app also includes a feature for users to concisely record self-measured parameters such as body weight, blood pressure, pulse rate, and body temperature as a diary function. The recorded values are displayed in a tabular format within the app, incentivizing users to actively engage with the app.

**Table 3:** Vital signs and physical activity parameters that will be recorded with the wearable

Parameter	Frequency	Unit
Raw photoplethysmography data (green, red, infra-red light)	32 Hz (up to 128 Hz)	
Heart rate	1/10 s	Beats per minute
Steps	1/10 s	Step count
Calories	1/10 s	
Core body temperature	1/10 s	°C
Respiration	1/10 s	Breath rate per minute (BRPM)
Peripheral oxygen saturation (SpO_2_)	1/10 s	%
RR intervals	1/s	ms
Sleep	1/30 s at nights	Categorical (1 = awake, 2 = rem, 3 = light, 4 = deep)
Bio impedance (BioZ)^a^	25 Hz	
ECG		No unit

^a^Explorative analysis only.

All study equipment will be collected at the end-of-study visit. In the event that no data is received from an enrolled patient for 1 day, the patient is reminded via a push notification to use the monitoring system. If no data is received for 2 consecutive days, the patient is contacted by phone to determine whether there is a technical problem. If the patient wishes to terminate the study, he will be excluded and asked to return the devices at the next regular visit to the study site. If this is not possible, the study team can offer to collect the devices at the patient’s home. If a patient is lost to follow-up, he will be contacted during the next regular visit to the study site to return the equipment.

### Data collection

Wearable data and ePRO data will be transmitted by monti via mobile data services to a centralized server that runs real-time data analysis and analysis software. The data recorded by the wearable will include time-series data of raw signals as well as vital signs and physical activity parameters, with the latter computed using proprietary algorithms provided by the manufacturer. Vital signs and physical activity parameters, such as heart rate, temperature, respiratory rate, and physical activity, will be recorded at a rate of up to 1 Hz. Thus, up to 3600 data points are potentially generated per hour, each paired by a corresponding quality index. On the platform backend, data integrity is assessed by monitoring actual wearing time, both initial and ongoing app usage, and overall data quality. Data integrity assessments are conducted at interim study visits and the study’s conclusion (*Table 2*).

Clinical records (visit entries, laboratory results, diagnostic results) will be documented in worksheets. Records will be independently and retrospectively reviewed by two cardiologists for the presence of ADHF. Worksheet entries and results of the validated questionnaires are transferred into a database.

Time-series data recorded by the wearable will be annotated based on clinical records. At each follow-up visit, where a clinical congestion assessment is conducted, the 2 days prior and post-visit are annotated as either ADHF or regular, as determined by the congestion assessment on the visit day.

Exploratively, the dataset of regular episodes may be enriched by including episodes beyond this initial 2-day window if patients report stable symptoms and vital signs through the app. This approach allows for the evaluation of whether these additional episodes show consistent ‘regular’ patterns suitable for model training. Datasets will be split into hours according to their timestamps, and only hours with a >90% amount of data points will be included to ensure sufficient information content among hours. No predefined quality constraints are used.

To calculate the risk index, ‘regular’ hours collected during the study are used to train a deep neural network with a self-supervised contrastive learning objective, extracting features typical of regular periods. Training and test data are split 90/10. The model is used to calculate a risk index that measures the dissimilarity to regular features. The detection and predictive performance of the risk index will be compared to clinical documentation of ADHF. To evaluate the risk index’s predictive capabilities over time, the timestamp of the clinical ADHF diagnosis will be used as a reference point. The risk index computed by the model will subsequently be examined for the days preceding an ADHF event. This analysis aims to identify early deviations in the risk index that could potentially be driven by subclinical changes in physical activity or vital sign patterns.

Data collection will be conducted following an intention-to-treat approach and will continue until withdrawal, except in cases where there is a specific request to delete all individually recorded data.

### Outcome and objectives

The primary outcome of this pilot study with the monti platform will be the accuracy of detecting episodes with diagnosed ADHF by a self-supervised contrastive learning-derived risk index based on wearable-recorded time series assessed through *C*-statistics (*Table 4*). As a secondary outcome data integrity will be assessed through the percentage of wearable data per day, the time difference between logged time point of measurement and database, and the score in the SUS, will be evaluated. Additionally, several exploratory endpoints will be examined.

**Table 4:** Objectives and outcome measures of the CONAN study

	Outcome measures
Primary objective	
Assess the capability of the self-supervised contrastive learning-derived risk index (from wearable-recorded time series) to detect episodes of acute decompensated heart failure (ADHF).	Area under the receiver operating characteristics (AUROC) curve for detecting episodes of diagnosed ADHF.
Secondary objective	
(2) Evaluate the data integrity of the *monti*-monitoring platform in terms of adherence, technical functionality, and data quality.	Adherence: Daily percentage of wearable data availability. Percentage of data availability over participation period. Availability of each parameter per day. Number of app-server connection attempts. Number of uploaded vital signs. Number of recorded ECGs. Number of dropouts. Technical functionality: Ratio of failed uploads to total uploads. Number/type of notifications sent via the app. Number/type of user contacts. Mean time difference between measurement and database logging. Data quality: Mean percentage of data with high quality indices determined by the wearable for each parameter per day.
(3) To assess usability of the mobile *monti* platform	Mean score on the System Usability Scale (SUS). Percentage of users rating the app as ‘easy to use.’
(4) Monitor adverse events, including device-related effects and deficiencies.	Number of skin reactions or discomfort. Number of observed device damages.
Explorative objective	
(5) Expansion of the dataset by including additional ‘regular’ episodes	Number of ‘regular’ episodes included for training, annotated based on the 2-day window of study visits. Number of the additional ‘regular’ episodes included for training, annotated based on patient-reported stable symptoms and vital signs via the app. AUROC detecting episodes with diagnosed ADHF with expanded training data.
(6) Assess detection capabilities using pre-trained neural networks.	Number of ‘regular’ episodes used for training. AUROC detecting episodes with diagnosed ADHF using pre-trained neural networks.
(7) Conduct subgroup analyses based on patient characteristics: sex, age, digital health literacy, quality of life (KCCQ-12), self-care behaviour (EHFScB-9), and inpatient vs. outpatient settings.	Area under the receiver operating characteristics (AUROC) curve for detecting episodes of diagnosed ADHF. Dropout rate. Percentage of wearable data availability over participation. Daily app-server connection attempts.
(8) Assess the robustness of clinical congestion assessments.	Correlation of congestion ratings based on clinical assessment with EVEREST Score. Correlation of clinical assessments with app-integrated symptom inventory.
(9) Evaluate the usability of the mobile *monti* platform using alternative scales.	Mean score on the Mobile Application Rating Scale (MARS-G). Correlation between SUS and MARS-G scores.
(10) Compare bioelectrical impedance analysis to clinical congestion assessments.	Correlation of clinical congestion assessment with total and extracellular body water (via bioelectrical impedance). Correlation of bioelectrical impedance analysis with wearable-measured bioelectrical impedance.
(11) Assess the *monti* platform impact on patient self-management, and quality of life.	Change in EHFScB-9 score (baseline to Day 90). Change in KCCQ-12 score (baseline to Day 90).

### Statistical analysis

The sample size calculation was based on a 20% incidence rate of congestion, an 85% detection sensitivity of the risk index (see below), a 15% dropout rate, a 10% error tolerance, and a significance level of 0.05, indicating a need for ∼282 patients to achieve sufficient statistical power.

For statistical analysis differences between means of hours annotated as ‘regular’ and ‘congested’ obtained from risk score will be tested for significance using a two-sided *t*-test test, adjustment for multiple comparisons is performed by using Bonferroni correction. To address overfitting, a 10-fold cross-validation will be performed. Statistical significance will be tested by an analysis of variance within the cross-validation splits of ADHF and regular. Receiver operating characteristics analysis [area under the curve of the ROC-analysis (AUROC)] will be computed to assess the primary endpoint. The cut-point that optimizes the detection of true positive results (sensitivity) and false positive results (1-specificity) is reported by the Youden index. For clinical requirements (not missing an event), specificity will additionally be reported at a sensitivity of ∼95%.

To assess risk score prediction capabilities for congestion detection, the performance of the score up to 7 days before and after the timestamp of diagnosis (t = 0 h) will be analysed. For AUROC-analysis, 95% confidence intervals are reported. A *P*-value of <0.05 will be considered significant. For data and statistical analysis, an open-source software tool will be used.

## Results

As of April 2027, the planned recruitment target should be reached. Data collection is expected to be completed by June 2027. The final study results are expected to become available within 6 months after study completion.

## Discussion

By performing this prospective observational cohort pilot study, we aim to assess the functionality and usability of a passive monitoring platform to detect ADHF in a cohort of patients with a history of ADHF for a duration of up to 3 months.

The primary outcome of this study is to assess the accuracy of a risk index in detecting ADHF. To account for individual variations in vital signs and physical activity, our self-supervised contrastive learning-derived risk index aims to detect divergence from a regular episode in the multiple parameters recorded, instead of focusing on a single parameter with rigid thresholds. A prerequisite for achieving high detection rates with this approach is the availability of a robust dataset of ‘regular’ episodes that enables reliable pattern recognition from wearable-recorded vital signs and physical activity parameters.

To support this, we defined a 2-day window around study visits for the annotation of regular episodes, ensuring a substantial dataset for training. Given the close temporal proximity of this window, significant differences in congestion state are considered unlikely. Additionally, the model must capture temporal patterns of wearable-recorded parameters under varying conditions, such as rest and physical activity, and in diverse settings, including inpatient and outpatient contexts. Achieving comprehensive coverage of these patterns—referred to as ergodicity—is anticipated to enhance the accuracy of ADHF detection. Conversely, insufficient ergodicity during these periods may diminish the informativeness of the risk index, potentially leading to an increased rate of false positives.

An exploratory approach will involve extending the ‘regular’ dataset by including episodes beyond the 2-day window, contingent upon patients reporting stability in symptoms and vital signs via the app. This evaluation aims to determine whether such episodes show consistent patterns that can enrich the dataset and improve ergodicity.

Furthermore, we will investigate the use of pre-trained neural networks, commonly referred to as Foundation Models, which are designed for specific applications such as image recognition. These models may optimize the pattern recognition of regular episodes, potentially reducing the amount of data required for effective training.^22^

By comparing against different congestion assessment tools, the performance and robustness of our risk index will be assessed.

The secondary endpoints are the assessment of data integrity and the potential for real-time monitoring. This includes technical parameters logged by the platform i.e. connection attempts to the server, as well as ePRO recorded with the app. Deficiencies of the monitoring system are systematically documented and analysed, whether they are technically or induced by patient handling. Thus, scenarios are identified, where insufficient data is available to potentially address those in upcoming studies. The patients’ perspective on such a monitoring system is crucial for real-world implementation; hence, the data obtained with the SUS and MARS-G questionnaire will be analysed at the study’s conclusion. The results of this analysis will be used to evaluate the longitudinal technical user-friendliness of the platform. Subsequently, recommendations for iterative system optimization will be formulated. By design, the focus of the development of this platform was to facilitate longitudinal monitoring to support patients with HF while minimizing disruption to their daily routines. This aspect will be thoroughly explored as part of the investigation.

Exploratory endpoints will evaluate whether the results from questionnaires assessing self-care, quality of life, and digital health literacy (EHFScB-9, KCCQ-12, and HLS19-DIGI) administered during the study correlate with patient adherence to study procedures and dropout rates. The data from these questionnaires, or specific dimensions within them, could potentially serve as screening tools in the future to identify suitable populations for wearable-based monitoring systems.

An additional aim of the study is the inclusion of patients with various HF entities and different congestion mechanisms, which may impact the reliability of the risk index. Nonetheless, this diversity allows us to assess whether a broad application among various mechanisms of decompensation of such a monitoring platform is applicable. Subgroup analyses considering e.g. sex, age, digital health literacy, quality of life, and self-care behaviour in HF will be performed to identify which patients may derive the greatest benefit from such monitoring approaches.

### Limitations

A limitation of the study is that the reliability of this risk index relies on the robust pattern recognition of regular episodes by high quantity and quality of the training data. Challenges include the continuum of clinical congestion from subclinical congestion without symptoms to full symptomatic presentation. Clinical congestion is not a binary condition and clinicians may incorrectly rate congestion. Therefore, different approaches to diagnose and grade clinical congestion are assessed.

By using the provided smartphone for data transfer, patients will likely leave the smartphone at home, which leads to a delayed transfer of the study data, when the wearable has no connection for data transfer. However, a provided smartphone is mandatory due to challenges such as data privacy and security concerns.

To evaluate the clinical benefit of a remote monitoring solution, a randomized controlled study has to be considered as the gold standard that provides stronger evidence. Such a study design will be the next step in determining the outcome benefits of such an approach for patient care. However, the study protocol presented in this manuscript has been conceptualized as a functionality and usability study before further research on comparing outcome benefits.

## Conclusion

In conclusion, this pilot study aims to evaluate the following key aspects to consider before a potential integration of a remote monitoring platform into a clinical care pathway.

First, the efficacy and safety of the monitoring platform in the pre-emptive detection of congestive episodes among a large cohort of patients with HF will be evaluated.

Secondly, a patient perspective is added through quality assessment of the app using the SUS questionnaire conducted under real-world conditions, as it is pivotal for the development of a widely applicable digital tool in HF management. This holistic approach will enhance technical understanding and is essential for the subsequent seamless integration into the clinical routine.

Thirdly, a successful risk index in the context of heart failure may serve as a valuable tool throughout the patient journey to monitor aspects such as treatment success.

This study will analyse the entire process, from data recording to the provision of a risk index for the detection of ADHF in patients with a history of ADHF as actionable information including technical and human factors.

## Data Availability

Data available on request.
